# The impact of face coverings on audio-visual contributions to communication with conversational speech

**DOI:** 10.1186/s41235-024-00552-y

**Published:** 2024-04-23

**Authors:** I. R. Jackson, E. Perugia, M. A. Stone, G. H. Saunders

**Affiliations:** 1 Manchester Centre for Audiology and Deafness, School of Health Sciences, University of Manchester, Manchester, M13 9PL UK; 2 Manchester Academic Health Science Centre, Manchester, UK

**Keywords:** COVID-19, Effort, Face coverings, Face masks, SARS-CoV-2, Speech intelligibility, Speech perception

## Abstract

The use of face coverings can make communication more difficult by removing access to visual cues as well as affecting the physical transmission of speech sounds. This study aimed to assess the independent and combined contributions of visual and auditory cues to impaired communication when using face coverings. In an online task, 150 participants rated videos of natural conversation along three dimensions: (1) how much they could follow, (2) how much effort was required, and (3) the clarity of the speech. Visual and audio variables were independently manipulated in each video, so that the same video could be presented with or without a superimposed surgical-style mask, accompanied by one of four audio conditions (either unfiltered audio, or audio-filtered to simulate the attenuation associated with a surgical mask, an FFP3 mask, or a visor). Hypotheses and analyses were pre-registered. Both the audio and visual variables had a statistically significant negative impact across all three dimensions. Whether or not talkers’ faces were visible made the largest contribution to participants’ ratings. The study identifies a degree of attenuation whose negative effects can be overcome by the restoration of visual cues. The significant effects observed in this nominally low-demand task (speech in quiet) highlight the importance of the visual and audio cues in everyday life and that their consideration should be included in future face mask designs.

## Introduction

Face coverings are an important tool for combatting transmission of aerosol-related infections (Chu et al., 2020; Howard et al., 2021). They are also widely used to reduce human exposure to particulates and other noxious substances. Widespread use of face coverings during the COVID-19 pandemic brought attention to the negative impacts of face coverings on communication (reviewed in Oosthuizen et al., 2022). By restricting access to auditory and visual cues conveying both information and emotion, face coverings can disrupt effective communication. Specifically, by covering a significant area of the lower portion of the face, masks not only remove many of the visual cues relied upon for speech reading (a superset of lip reading), but also affect the physical transmission of speech sounds that reach a listener. Further impacts include increased difficulty disambiguating subtleties in communication, such as the use of emotion expressed through the face (McCrackin et al., 2022; Rinck et al., 2022). The cumulative disruption makes communication more difficult for all, but particularly for individuals who already face challenges in communication, such as people with hearing loss (Saunders et al., 2020; Tavanai et al., 2021).

Different types of face covering attenuate the acoustic signal to varying degrees. Generally, for speech, face coverings effectively operate as a ‘low-pass filter’. Speech signals are relatively unaffected up to around 1 kHz, but become increasingly attenuated at higher frequencies (Atcherson et al., 2021; Corey et al., 2020; Cox et al., 2022; Pörschmann et al., 2020; Rahne et al., 2021). In good listening conditions (e.g. low levels of background noise and clear speech), the effects of face coverings on speech understanding can be minimal, even for the hearing impaired (Mendel et al., 2008; Vos et al., 2021). As background noise levels increase; however, effects on speech understanding become increasingly detrimental, and the functional effect of different types of face covering become more pronounced (Brown et al., 2021; Carraturo et al., 2021; Mendel et al., 2008; Toscano & Toscano, 2021). Even when objective performance in speech understanding tasks is similar across conditions with and without face coverings, the use of face coverings nonetheless increases subjective ratings of listening effort (Brown et al., 2021).

Behavioural adjustment by speakers is one strategy used to compensate for the effect of face coverings on communication. Such adjustments can include speaking more loudly, more slowly, and more clearly (Gutz et al., 2022). Indeed, these and other adjustments, such as introducing longer pauses into speech, appear to be commonly and spontaneously adopted by talkers when wearing face coverings (Cohn et al., 2021; Magee et al., 2020; McKenna et al., 2022). Our group has previously conducted research into participants’ subjective perceptions of changes in communication when using face coverings (Saunders et al., 2020). In that work, participants reported that face coverings changed the quality of interactions, leading them to engage in less complex, deep, and spontaneous conversation, and also that masks led to decreased interpersonal connection. Compensatory behaviours, however, are effortful and tiring over prolonged use (Gutz et al., 2022; Ribeiro et al., 2020; Shekaraiah & Suresh, 2021). A more optimal (or complementary) solution would be to improve face covering design to reduce the degree of effort required by both speakers and listeners.

One commonly suggested design improvement is the introduction of transparent panels, which provide listeners with increased visual access to a talker’s mouth and face (Atcherson et al., 2017; Corey et al., 2020; Cox et al., 2022; Tavanai et al., 2021; Thibodeau et al., 2021; Yi et al., 2023). The benefit of access to visual cues on performance in speech understanding tasks is well established, including evidence to support the benefit of transparency in face masks (Atcherson et al., 2017; Erber, 1969; Giovanelli et al., 2021; Llamas et al., 2008; Macleod & Summerfield, 1987; Sönnichsen et al., 2022; Sumby & Pollack, 1954; Thibodeau et al., 2021; Yi et al., 2021, 2023). In a speech recognition task, Thibodeau et al. (2021) presented participants with videos of a speaker wearing a mask containing a transparent window, and videos of the speaker wearing the same mask but with a piece of fabric covering the transparent section (blocking visual access to the speaker’s mouth). Speech signals were presented in background noise with a − 5 dB signal-to-noise ratio (SNR) and the difference in attenuation between the two conditions was minimal. (Overall attenuation was 1 dB greater for the transparent mask condition, relative to no mask at all.) Performance was significantly better for the condition where the speaker’s mouth could be seen; a mean of 69% correct when the speaker wore a transparent mask, compared to 59% for the opaque mask. When the speaker was not wearing any mask at all, speech recognition was 84% correct, significantly more accurate than in either of the mask conditions. Atcherson et al. (2017) compared performance on a speech perception task when a speaker wore a paper mask, a transparent mask, or no mask at all. Stimuli were presented in the presence of background noise with an SNR of + 10 dB. They found that participants with normal hearing performed consistently well across all conditions, whether visual cues were available or not. Speech perception for participants with hearing loss, however, was significantly improved when audio-visual recordings of transparent face coverings were presented, relative to audio recordings made with masks and where no visual information was provided to the listener.

These studies each examined speech performance using standard tasks. Speech intelligibility tasks, however, capture only limited aspects of natural communication in real-world contexts and do not examine factors such as required effort, or fatigue during task performance (e.g. Beechey, 2022; Winn & Teece, 2021). In an online experiment, Giovanelli et al. (2021) showed that removing visual cues from listeners resulted in significantly lower performance in a speech-in-noise comprehension task, lower confidence in responses, and increased perceived effort. In addition to improvements on measures of speech intelligibility, a wider benefit of transparent panels is seen in alternative measures of communication success such as emotion recognition, whose variety is conveyed by the full face rather than just the lips and teeth (Wegrzyn et al., 2017).

The benefits of improved access to visual cues are clear. Transparent panels and visors, however, are also associated with increased attenuation of the acoustics of the speech signal, unless specially designed (Cox et al., 2022). The materials typically used for transparent panels have been stiffer but also less absorptive than the non-transparent fibrous materials used for filtration, leading to both resonances and absorption in sound transmission. Hence, a trade-off exists where the visual gain of a transparent panel is offset by both acoustic gains and losses (Cox et al., 2022). In the Thibodeau et al.’s (2021) study discussed above, speech recognition was significantly better for transparent masks compared to opaque masks. In a follow-up study in the same paper, listeners were presented with the same speech signals but were provided with no visual cues. In this auditory-only condition, the original findings were reversed; performance was significantly better for recordings made with the opaque mask (58% correct) compared to the transparent mask (40%). In other words, performance for the opaque mask was unaffected by whether or not video of the speaker was available. For the transparent mask however, the significant drop in performance in the acoustic-only condition demonstrates both the importance of visual cues for speech recognition, as well as the negative acoustic effects of transparent panels on speech transmission. Improved communication between wearers of face coverings could be achieved with improved face covering design (Cox et al., 2022). Greater understanding of the nature of the trade-off between increased visual access to speakers’ faces and acoustic losses associated with the materials used in transparent masks is key to informing the design process for improved mask designs, as well as developing improved strategies for communication.

Two recent studies have examined elements of the relative trade-off. Sönnichsen et al. (2022) tested 15 healthy-hearing adults in an intelligibility task featuring a single, female speaker presented in background noise. At a threshold SNR where participants correctly recognised 80% of the words presented to them, they found independent and additive effects for both visually- and audio-simulated use of face coverings. Both effects were of a similar magnitude; a 2.5 dB SNR loss for audio simulation of cloth mask versus 2.6 dB SNR loss for visual information of using a mask (corresponding to a difference in speech intelligibility of about 30% from a no-mask baseline in each case). Giovanelli et al. (2021) manipulated the balance between audio and visual cues in a task involving a simulated online video-conference call. In this work, four talkers (1 male, 3 females) provided individual audio-visual recordings of themselves uttering fixed-format short sentences (the Italian matrix test). These audio-visual recordings were then arranged into a 2 × 2 video grid. In the grid, sentences from the male talker were presented in a background noise of similar sentences produced by either one or all three of the female talkers. The audio accompanying each condition was that recorded by speakers with no mask, thus isolating the effect of visual manipulation on speech intelligibility. The number of correctly reported words, participants’ confidence in their responses, and degree of listening effort were all significantly poorer in the conditions in which faces were partially or fully occluded.

### The current study

To date, the majority of studies on the effects of face coverings on communication focus on measures of intelligibility (see Badh & Knowles, 2023, for a review). Given the wider impacts of face coverings on naturalness of communication, such as reviewed in Oosthuizen et al. (2022), our focus in the current work was on the relative impacts of visual and audio cues on natural, real-world conversation. Like Giovanelli et al. (2021), we explored manipulations of audio-visual cues in a video-conference call using an online task. Unlike that study, however, which made use of a highly structured format and artificially constructed the appearance of a video-conference call, we presented participants with actual screen-recordings of unscripted conversation between friends in a real video-conference call. We assessed the effect of both an auditory and visual manipulation. For the visual manipulation, a surgical-style mask was digitally superimposed over the faces of those on the video-conference call. For the audio manipulation, rather than the commonly used approach of adjusting the level of background noise, we presented the soundtracks in quiet but simulated the attenuation associated with different types of face covering. Using this approach allowed for the independent and joint assessment of the effect of visual and audio cues associated with a variety of commonly used face coverings. The soundtracks were much longer in duration than single sentences to allow for the development of the real dynamics of conversation.

We designed the study to test three pre-registered hypotheses (Jackson et al., 2022), which can be summarised as:

- *Hypothesis 1:* Relative to an unfiltered condition, ratings of (1) the ability to follow the conversation, (2) the effort required, and (3) how clear speech was perceived to be, will be more negative in conditions where the audio has been filtered to simulate the acoustic effects of different types of face covering, and ratings will become more negative as the degree of filtering increases.
- *Hypothesis 2:* Digitally superimposing a face covering over a speaker’s mouth will negatively impact ratings of (1) the ability to follow the conversation, (2) the effort required, and (3) how clear speech was perceived to be, relative to not superimposing a face covering.
- *Hypothesis 3:* There will be an interaction between the audio filtering and visual superimpositions such that the negative impacts of the audio filtering and superimposition of face covering will be greater than the sum of the individual impacts.

## Methods

### Participants

A total of 150 participants (101 females, 46 males, 3 preferred not to say) completed the experiment online. The mean age of the participants was 36.2 years (SD = 13.8 years, range = 18–84 years). Seventy-four per cent (i.e. 111 participants) reported that English was their first language. Data collection took place between May and July 2022. The study was approved by the University of Manchester proportionate Research Ethics Committee (ref: 2021-12348-20345).

On a five-point verbal scale (poor, fair, good, very good, excellent), 90% of participants reported their hearing as being ‘good’ or better. Six participants (4%) reported that they had at least one hearing aid. Of those 6 participants, 4 reported that they were wearing their hearing aid(s) during the experiment. Using the same five-point scale as for hearing, 98% reported that their vision was ‘good’ or better. Ninety-three participants (62%) reported that they wear glasses or contact lenses when using a computer, of whom 90% (i.e. 84 participants) reported wearing them for the current task.

Participants generally had experience with video-conferencing tools. Eighty-one per cent (i.e. 121 participants) reported using online communication platforms (e.g. Zoom, Teams, etc.) at least a couple of times per week or more. Only two participants reported that they ‘never or almost never’ used online communication platforms.

### Stimuli

**Fig. 1:**
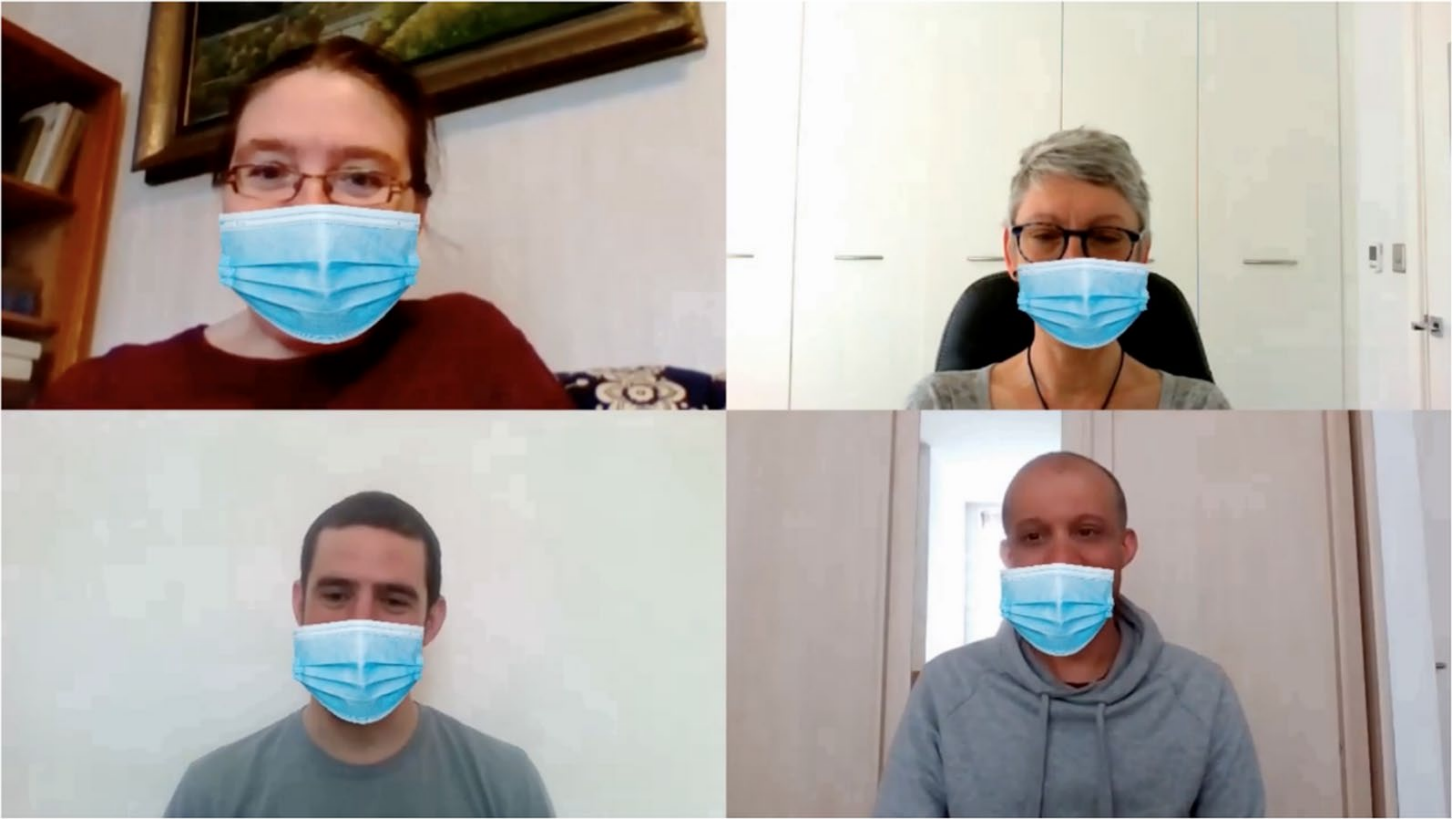
Composite figure of the four participants ‘wearing’ computer-imposed face coverings, assembled from the sub-images in a single frame of a recording of a four-way Zoom conversation

Stimuli were clips selected from continuous screen-recordings of natural, conversational speech in a Zoom teleconference call (Zoom Video Communications Inc, 2022) between four friends (authors IJ, EP, GS, and one other; two males and two females). The friends all spoke fluent English. Of the four, one woman and one man spoke English as their first language, the second woman spoke German as a first language, and the second man spoke Italian as a first language. See Fig. 1 for a screenshot.

The screen-recordings were edited to explore two intertwined experimental manipulations: a visual component and an auditory component. In the visual manipulation, we controlled whether or not viewers could see the speakers’ mouths by digitally superimposing an image of a surgical-style mask onto each face on the Zoom call. In the auditory manipulation, the audio from the video recordings was filtered to simulate the attenuating effect of each of three different types of face covering. This approach allowed us to assess the impact of the visual and auditory components independently and jointly, while also controlling for the conversational content and non-experimental perceptual features of each video recording. (Examples of the different conditions can be played in the supplementary materials.)

The recorded conversation centred on multiple rounds of the game ‘20 Questions’. In this well-known guessing game, one person thinks of an object, animal, or similar, and the other players must guess what it is. The guessers take turns to ask questions which can be answered only with a “yes” or “no” response. The aim of the game is for the guessers to identify the object within 20 questions.

Multiple rounds of the 20 Questions game took place, with the people on the call taking turns over who thought of the object for the next round. The full screen was recorded throughout. The game was played spontaneously, with no pre-planning of rounds and no practice. Once completed, eight rounds of the game were selected from the continuous recording. Each person on the call contributed two rounds to the eight selected. Each round was then split into 8 segments of roughly equal duration. Segments were not exactly equal in duration as some variation was necessary to maintain the natural breakpoints in conversation (e.g. to ensure that that talkers were not cut-off mid-sentence). Mean duration of segments across all questions was 19.6 s (SD = 3.5 s).

### Audio-visual manipulations

**Fig. 2:**
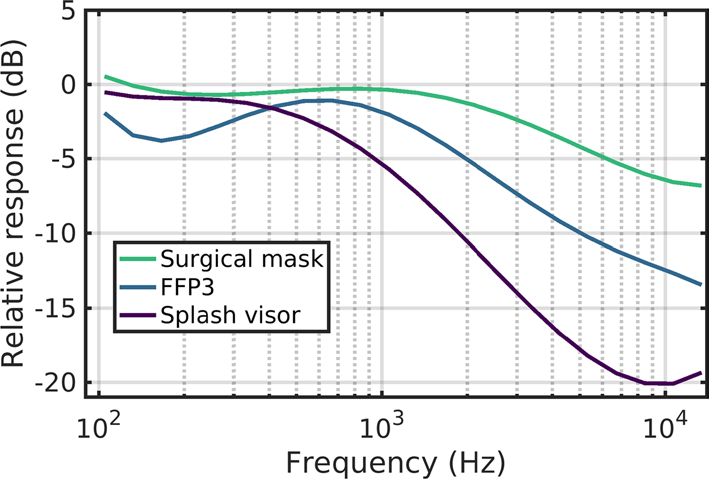
Attenuation characteristics for each of the three types of face covering simulated in the test

Audio from the recordings was then filtered to produce three audio conditions per segment, simulating the acoustic attenuation associated with (1) a surgical mask, (2) an FFP3 mask (equivalent to N99 in USA), and (3) transparent mask/visor. A fourth audio condition, the unfiltered audio recordings from the video-conference call, was also included in the test set for each segment of video. Audio filtering was performed in MATLAB (MathWorks, 2019). Acoustic filters used for processing were based on the acoustic attenuation measures reported in Munro and Stone (2020). In that work, acoustic transmission measures of four face coverings were reported as measured in a near-anechoic room. Since two of their measures were from a similar level of mask filtration ability (FFP3, either fold-flat or pre-formed), we averaged their near-identical attenuations to produce a single simulation of that style. Additionally, we used their measures for the surgical mask and the transparent visor, with, respectively, a lesser and greater degree of attenuation when compared to the FFP3. The attenuation responses with frequency used here were similar in degree to other reported measures for face coverings (Atcherson et al., 2021; Corey et al., 2020; Cox et al., 2022), but were smoothed to reduce the ‘lumpiness’ visible in those reports. This lumpiness appears to be dependent on fine details of the mask design, and possible interactions with the skull for masks, and body for visors, leading to resonances. The attenuation characteristics we used should therefore be seen as gradations of severity, from mild, through moderate to severe (average attenuations in the region 2–10 kHz of − 3, − 8 and − 16 dB, respectively). Figure 2 illustrates the attenuation characteristics for each of the three types of face covering.

All recording, analysis, filter generation, and filtering was performed at a sample rate of 44.1 kHz. Audio segments (including the clean, raw audio recordings from the video-conference call) were normalised to the same level using root-mean-square (RMS) normalisation for presentation in the test. Extended details of the filtering process can be found in the supplementary materials.

To create the manipulation in the visual component, a duplicate copy was made of all videos used in the experiment. In the duplicate copies, an image of a surgical-style mask was digitally superimposed over the face of each person on the call. The position of the mask on each person’s face was partially automated using the Track Motion feature in Corel VideoStudio X10 (Alludo, 2017) and then manually fine-tuned frame-by-frame as necessary.

A complete set of stimuli was then generated to create every possible permutation of audio and visual conditions for each segment. This full set comprised 512 video stimuli:

8 questions (2 rounds of the game per each of the four people in the Zoom call)

* 8 segments per question.

* 2 visual conditions (no mask vs superimposed surgical mask).

* 4 audio conditions (unfiltered audio vs surgical simulation vs FFP3 simulation vs visor simulation).

There were 8 possible permutations of the visual (2 levels) and audio (4 levels) variables. Each question was divided into 8 segments, and one permutation was randomly assigned to each segment. Thus, across the 8 segments in a question, participants were presented with all possible combinations of the experimental conditions. Participants completed two of the eight possible questions per experiment. Questions were randomly assigned.

The randomisation patterns were pre-calculated by creating a lookup table of 400 possible permutations. After consenting to take part, each participant was randomly assigned to one permutation (without replacement). Although the maximum number of participants was capped in advance at 150, an excess of permutations was created to allow for an unknown number of participants who would start the experiment but not go on to complete it.

Our goal with the visual manipulation was to isolate the effect of visual cues and so a single mask type was used consistently across all conditions. It should be noted that, for completeness, the part of the experimental design simulating the visor acoustic effect included a ‘mask’ and ‘no-mask’ condition. During the COVID-19 pandemic, it was common for health professionals to wear a surgical mask (low attenuation) under a visor (high attenuation), so the ‘mask’ visual condition with the visor acoustic condition is still a realistic scenario.

### Choice of outcome measures

Self-report measures were used for both theoretical and practical reasons.

1. While objective measures of intelligibility offer precision in the context of structured, controlled, laboratory testing, they do not generalise well to real-world performance (Miles et al., 2022) and provide limited insight into wider aspects of communication (Baese-Berk et al., 2023).
2. Ceiling effects are common in intelligibility tasks when task demands are low (e.g. Atcherson et al., 2017; Mendel et al., 2008), and the content of speech used in formal measures of speech intelligibility is limited and unnatural. For the current task, in which sounds were presented in quiet, and anticipated experimental effects were modest, a standard intelligibility measure was unlikely to be sufficiently sensitive to provide useful discrimination between conditions.
3. Our focus on natural conversation in this study precluded the use of existing materials which rely on participants repeating back simple isolated low-context sentences. We therefore chose to examine subjective aspects of speech perception and communication that are representative of real-world conversation—namely how much of a conversation participants could follow, how much effort was required to follow the conversation, and how clear the speech was. Previous investigations of subjective effort with face coverings have used a mix of existing (Brown et al., 2021; Rahne et al., 2021) and ad hoc scales (Lee et al., 2022). None of these scales would have been suitable for the current study.
4. We wanted participants to make ratings of multiple short clips of speech and thus needed single rather than multi-item scales, that were consistent in terms of usage and appearance, and that used the same underlying numeric (rather than ordinal) scale.
5. The study was conducted online and thus use of physiological measures and measures that require calibration were not practical. Further, we wanted to minimise missing data, which commonly arises during online testing because of the ease with which participants can drop out of a test session.

In summary, we wanted the experiment to reflect real-world communication, be engaging, short in duration, and to have low-task demands. We thus opted for 3 single-item responses on each trial to capture understanding, effort, and clarity that used the same scale and response format.

### Procedure

The experiment was created and hosted using Gorilla Experiment Builder (www.gorilla.sc; Anwyl-Irvine et al., 2020). Participants were recruited via social media, mailing lists, and internal email announcements sent to University of Manchester staff.

Participants were requested to complete the task using a computer/laptop and headphones. The type of online device used to access the experiment was automatically screened by the host software, and access to the experiment was denied to those who attempted to take part using a mobile phone. Participants were also asked to specify the method of playback they used. Playback method fell roughly equally into one of three categories: built-in speakers on a computer or laptop (33%), earphones or earbuds (28%), or headphones (38%). Only two participants reported using something other than these categories, one who reported using “a sound system connected via Bluetooth”, and another who used “induction ear hooks linked to laptop”.

Before they were allowed to begin the test, participants had to prove they were a genuine responder, rather than a ‘bot’, by passing a simple visual identification task. Participants were then given some example audio before the test began and were requested to set the volume of their playback device to a comfortable level. Only those participants who completed the task in full contributed data to the final sample. Participants who accessed the task but did not complete it within 2.5 h were automatically rejected.

In the task itself, participants manually started each video clip by clicking on a play button. Clips could only be played once.

The impact of the audio and visual variables was assessed using participant ratings on three related, but distinct, subjective aspects of speech perception. After each video participants provided three ratings by manipulation of a graphical slider:

1. “Approximately how much of what was said could you follow?” Slider endpoints were “0% (Nothing)” and “100% (Everything)”.
2. “How much effort was it to understand what was being said?” Slider endpoints were “Little or no effort” and “A lot of effort”.
3. “How clear was the speech in the clip?” Slider endpoints were “Very unclear” and “Very clear”.

Rating (1), the amount of conversation participants could follow, taps into real-world estimation of intelligibility, rather than, for example, individual word scores under artificial constraints in the laboratory. A recent review (Baese-Berk et al., 2023) highlights how “measures [of intelligibility] alone fail to capture the complexities of speech perception, suggesting that other tools, methodological and statistical, will provide more insight into the processing challenges faced by listeners in many real-world settings”.

Rating (2), the amount of effort required, is distinct from how much was followed in that it is possible to follow two different talkers equally well, but following one talker’s speech may require considerably more effort than the other.

Finally, rating (3), how clearly the speech was perceived, is also a related but distinct outcome; two examples of speech could be considered equally easy to follow, and equally effortful, but nonetheless recognisably different in clarity.

The rating scales on the screen were unmarked, other than with the endpoints described above. The underlying numerical values for each scale ran from 0 to 100 in integer increments. The starting position for each scale was the midpoint of the scale, and participants could not progress to the next screen without having moved each slider (once moved off-centre, the slider could then be positioned back to its original midpoint location).

After providing ratings on each trial, participants were presented with the multiple-choice question “Who was the last person to speak in the clip you just saw?” along with images and the names of the four people on the call (pseudonyms were used in place of real names during the task). This was done to obtain information about each participant’s engagement with the task. Responses were submitted by clicking on one of four buttons showing the talkers’ names. In cases where the last person to talk was ambiguous (for example whether a laugh or an “ahh”, “erm”, “hmm” should be considered to be speech) responses were coded as correct either if participants identified either the last person to contribute actual speech (i.e. a whole word) *or* the last identifiable noise. Seventy-seven per cent of participants scored 12 or more out of a maximum of 16 correct responses, indicating strong engagement with the task.

On completion of the task, participants could opt to receive a £10 electronic voucher reward. Of the 150 participants, 138 requested a voucher. The mean time taken to complete the experiment was 17 min. Extended details and screenshots from each stage of the experiment are provided in the supplementary materials.

### Data processing and analysis

Data from all participants were analysed; no exclusions applied at either the participant or trial level. Separate linear mixed-effects models were used to assess the effects of the visual and auditory variables on each of the three ratings scales: (1) the amount of conversation followed, (2) the amount of effort required to understand the conversation, and (3) the clarity of speech heard. Predictor variables were treatment-coded, with ‘No mask’ as the reference level for the visual variable and with ‘Clean’ as the reference level for the auditory variable.

The initial starting point for all models was the pre-registered full model, which consisted of an interaction between the auditory and visual variables in the fixed effects, as well as by-item and by-participant random slopes and intercepts for the interaction. Complexity of models was iteratively reduced until they converged, first by removing the interaction term from the random effects structure, then by removing whichever random slope term contributed least variance to the model at each subsequent step. Code and results for each intermediary step are available in the project’s open materials.

Our maximal model for each measure was specified as follows:

- lmer(response ~ 1 + 
- audio_condition * visual_condition + 
- (1 + audio_condition * visual_condition | participant_id) + 
- (1 + audio_condition * visual_condition | item))

All data processing and analysis took place in R (R Core Team, 2022), using the *Tidyverse* family of packages (Wickham et al., 2019), and the *lme4* (Bates et al., 2015) package for mixed-effect model analyses. Effect sizes were estimated using the *effectsize* package (Ben-Shachar et al., 2020).

All analyses and figures are fully reproducible using the openly available code and de-identified data in the online repository for the project, which can be found at https://osf.io/r9tmh/. MATLAB code for performing the filtering used in the audio simulations is also available in this repository.

## Results

### Confirmatory analyses

1. “How much of the conversation could you follow?”

The final model used for analysis of this measure was:

- lmer(response ~ 1 + 
- audio_condition * visual_condition + 
- (1 + visual_condition | participant_id) + 
- (1 | item),
- control = lmerControl(optCtrl = list(maxfun = 2e + 5)))

A statistically significant interaction between the audio and visual variables was observed (*p* = 0.042, *η*_*p*_^2^ = 0.004), as well as statistically significant simple effects of both the audio (*p* = 0.002, *η*_*p*_^2^ = 0.009) and visual variables (*p* < 0.001, *η*_*p*_^2^ = 0.205) independently. For the visual variable, as predicted, less of the conversation was followed when participants were wearing face coverings than when faces were fully visible (*β* = − 4.57, 95% CI [− 6.80, − 2.35], *p* < 0.001). The hypothesis for the audio variable was only partially supported. Ratings for the surgical mask and FFP3 mask simulations were not statistically significantly different from those for the clean audio (*β* = − 0.66, 95% CI [− 2.66, 1.33], *p* = 0.51; *β* = 0.16, 95% CI [− 1.83, 2.15], *p* = 0.87, respectively), but less of the conversation was followed when the accompanying audio-simulated use of a visor than when it was the original, clean audio (*β* = − 3.24, 95% CI [− 5.23, − 1.25], *p* < 0.001). Full results can be found in the model summaries shown in Table 1. (Expanded details can be found in the supplementary materials.)

**Table 1:** Model summaries for each of the three measures. Intercepts represent the mean of the reference level of each predictor variable (‘Clean’ for the auditory variable, and ‘No mask’ for the visual variable)

	Follow					Effort					Clarity				
	Beta	SE^1^	Statistic	95% CI^1^	*p* value	Beta	SE^1^	Statistic	95% CI^1^	*p* value	Beta	SE^1^	Statistic	95% CI^1^	*p* value
(Intercept)	88	1.28	69.0	86, 91	< 0.001	18	1.67	10.6	14, 21	< 0.001	84	1.54	54.8	81, 87	< 0.001
Audio condition					0.002					0.023					0.002
Clean	–	–	–	–		–	–	–	–		–	–	–	–	
Surgical	− 0.66	1.02	− 0.653	− 2.7, 1.3		− 0.37	1.31	− 0.280	− 2.9, 2.2		− 0.90	1.28	− 0.706	− 3.4, 1.6	
FFP3	0.16	1.02	0.158	− 1.8, 2.2		0.77	1.31	0.589	− 1.8, 3.3		− 1.1	1.26	− 0.878	− 3.6, 1.4	
Visor	− 3.2	1.01	− 3.19	− 5.2, − 1.3		3.3	1.31	2.52	0.72, 5.8		− 5.0	1.41	− 3.56	− 7.8, − 2.2	
Visual condition					< 0.001					< 0.001					< 0.001
No mask	–	–	–	–		–	–	–	–		–	–	–	–	
Mask	− 4.6	1.13	− 4.03	− 6.8, − 2.3		8.1	1.50	5.43	5.2, 11		− 7.4	1.33	− 5.60	− 10, − 4.8	
Audio condition * visual condition					0.042					0.81					0.13
Surgical * mask	1.5	1.44	1.02	− 1.3, 4.3		− 0.41	1.85	− 0.219	− 4.0, 3.2		0.60	1.67	0.361	− 2.7, 3.9	
FFP3 * mask	− 2.2	1.44	− 1.50	− 5.0, 0.67		1.1	1.85	0.615	− 2.5, 4.8		− 2.3	1.67	− 1.35	− 5.5, 1.0	
Visor * mask	1.3	1.44	0.924	− 1.5, 4.1		− 0.42	1.85	− 0.226	− 4.0, 3.2		1.6	1.68	0.940	− 1.7, 4.9	
Number of observations	2400					2400					2400				

^1^*SE* standard error, *CI* confidence interval

**Fig. 3:**
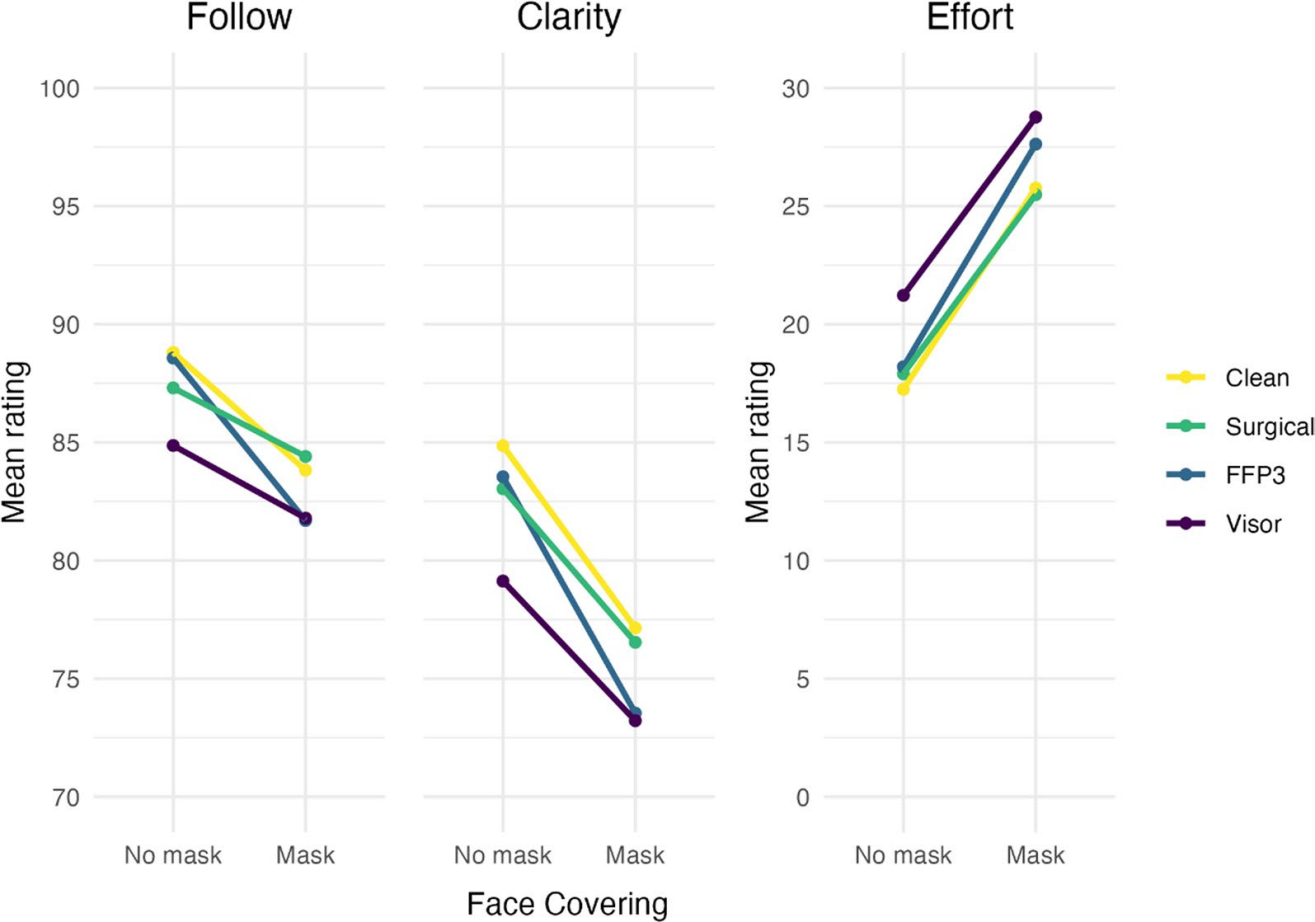
Mean ratings for each of the three outcome measures in separate panels; how much of the conversation participants could follow, how clear the speech was, and how much effort was required to follow the conversation. Data is split by the visual variable along the x axis, and the levels of the auditory condition are represented by individual lines on each panel. Note, *y* axes have been truncated for ease of interpretation (the range of the scale used to collect data was 0 to 100 for each of the three measures)

The statistically significant interaction between audio and visual variables is visualised in the “*Follow*” panel of Fig. 3. To decompose the interaction, we compared ratings for the visual condition (face covering vs no face covering) at each level of the audio variable, using Šidák corrections for multiple comparisons. As predicted, the introduction of a face covering leads to a statistically significant reduction in the amount of conversation participants could follow, regardless of which audio condition was presented (for the clean audio, *t*(810) = 4.03, *p* < 0.001; for the surgical mask, *t*(806) = 2.74, *p* = 0.025; for the FFP3, *t*(802) = 5.95, *p* < 0.001; and for the visor, *t*(809) = 2.86, *p* = 0.017). However, the relative decrement in following the conversation did not increase as expected with the level of attenuation, appearing instead more pronounced for the clean and, in particular, FFP3 audio conditions, relative to the surgical and visor conditions.

(2) “How much effort was it to understand what was being said?”

The final model used for analysis of this measure was:

- lmer(response ~ 1 + 
- audio_condition * visual_condition + 
- (1 + visual_condition | participant_id) + 
- (1 | item),
- control = lmerControl(optCtrl = list(maxfun = 2e + 5)))

Statistically significant simple effects were observed for the audio (*p* = 0.023, *η*_*p*_^2^ = 0.009) and visual variables (*p* < 0.001, *η*_*p*_^2^ = 0.324). The predicted interaction between the two variables was not significant (*p* = 0.81, *η*_*p*_^2^ < 0.001). For the visual variable, as predicted, understanding what was being said was significantly more effortful when talkers were wearing face coverings than when faces were fully visible (*β* = 8.12, 95% CI [5.19, 11.06], *p* < 0.001). For the audio condition, our hypothesis was only partially supported. Ratings for the surgical mask and FFP3 mask simulations were not statistically significantly different from those for the clean audio (*β* = − 0.37, 95% CI [− 2.94, 2.20], *p* = 0.78; *β* = 0.77, 95% CI [− 1.80, 3.34], *p* = 0.56, respectively), but significantly more effort was required to understand what was being said in the visor condition than in the clean audio condition (*β* = 8.12, 95% CI [5.19, 11.06], *p* < 0.001). Full results can be found in the model summaries shown in Table 1. (Expanded details can be found in the supplementary materials.) Mean ratings for effort are presented in the “*Effort*” panel of Fig. 3.

(3) “How clear was the speech in the clip?”

The final model used for analysis of this measure was:

- lmer(response ~ 1 + 
- audio_condition * visual_condition + 
- (1 + visual_condition | participant_id) + 
- (1 + audio_condition | item),
- control = lmerControl(optCtrl = list(maxfun = 2e + 5)))

Statistically significant simple effects were observed for the audio (*p* = 0.002, *η*_*p*_^2^ = 0.263) and visual variables (*p* < 0.001, *η*_*p*_^2^ = 0.344). The predicted interaction between the two variables was not significant (*p* = 0.13, *η*_*p*_^2^ < 0.003). For the visual variable, as predicted, speech was rated as being significantly less clear when talkers were wearing face coverings than when faces were fully visible (*β* = − 7.44, 95% CI [− 10.05, − 4.84], *p* < 0.001). For the audio condition, our hypothesis was only partially supported. Ratings for the surgical mask and FFP3 mask simulations were not statistically significantly different from those for the clean audio (*β* = − 0.90, 95% CI [− 3.42, 1.61], *p* = 0.48; *β* = − 1.11, 95% CI [− 3.58, 1.36], *p* = 0.38, respectively), but speech was perceived to be significantly less clear in the visor condition than in the clean audio condition (*β* = − 5.02, 95% CI [− 7.78, − 2.25], *p* < 0.001). Full results can be found in the model summaries shown in Table 1. (Expanded details can be found in the supplementary materials.) Mean ratings for speech clarity are presented in the “*Clarity*” panel of Fig. 3.

## Discussion

Visual access to the mouth and lower portion of the face is known to improve intelligibility in speech perception tasks (Preminger et al., 1998). Transparent panels in face coverings have the benefit of improving visual access to the face, but also come with the cost that transparent panels are typically less permeable to sound, and so can make speech more difficult for listeners to perceive. Informed decisions about the use and design of face coverings rely on a strong and nuanced understanding of the effects different materials and designs have on each aspect of communication, in combination with knowledge of the hearing abilities of the target population. The aim of the current study was to contribute to this understanding by systematically assessing the effect of the visual and auditory components, both independently and jointly, in a large, diverse sample.

We assessed three pre-registered hypotheses. The first, that ratings would be negatively impacted by the simulated attenuating effect of face coverings, was broadly supported. We observed a statistically significant effect of the audio variable for each of the three subjective measures; the attenuating effect of simulated face coverings resulted in significantly less of the conversation being followed, meant significantly more subjective effort was required by the listener, and made speech significantly less clear. The overall effect size of the audio manipulation was much larger for perceptions of clarity than for either how much of the conversation was followed or for how effortful it was to understand the conversation. This finding suggests that participants were reliably able to perceive a difference between the clean and attenuated audio conditions, even if the perceived difference did not necessarily result in large, negative consequences in the other two measures. We predicted that each type of face covering would be rated more negatively than the unprocessed audio (i.e. the clean audio in the no-mask condition). For each of the three subjective measures, it was broadly the case that ratings became more negative as the degree of attenuation increased, but only the simulated visor condition was associated with statistically significantly poorer ratings than the unprocessed audio condition.

The second hypothesis was that ratings would be negatively impacted by the addition of a superimposed surgical-style mask over the faces in the videos. This hypothesis was strongly supported across all three measures. Effect sizes in each measure suggest that the visual manipulation had a large effect in making it harder to follow conversations, more effortful to understand what was being said, and making speech less clear.

The third hypothesis was that an interaction would be observed between the auditory and visual variables we manipulated. This hypothesis was only partially supported. A statistically significant interaction was observed for how much of the conversation was followed, but not for how much effort was required or how clear speech was perceived to be. The observed interaction can be seen in the Follow panel of Fig. 3 through the relative impact of the visual variable on each of the different type of face covering; the addition of a surgical-style mask to the faces in the video made conversation harder to follow in all audio conditions, but the impact was relatively stronger for the unprocessed and simulated FFP3 audio than it was for simulated surgical and visor audio. The overall magnitude of the interaction, however, was very small.

In terms of the relative contribution of visual and auditory variables to communication difficulties in the quiet listening conditions we used here, our findings strongly suggest the removal of visual cues is more impactful than the acoustic degradations that face coverings can impose on speech transmission. Our goal in this study was to assess audio-visual contributions to communication in favourable listening conditions. Generally, under these conditions, we observed significant impairment only at the highest level of attenuation—that associated with our simulation of visors. Communication was relatively unaffected by the level of attenuation associated with surgical and FFP3 masks. This finding is consistent with previous studies investigating speech presented in quiet or relatively high SNR (i.e. favourable listening conditions), in which differences reported are very small or non-significant (Atcherson et al., 2017; Brown et al., 2021; Mendel et al., 2008), but become more marked as the level of background noise increases (Brown et al., 2021; Toscano & Toscano, 2021), especially for those with hearing loss (Atcherson et al., 2017; Ritter et al., 2022). In addition to speech intelligibility, Brown et al (2021) also used a rating of subjective listening effort. In that study, the use of transparent masks did not significantly affect intelligibility in quiet but did moderately increase subjective effort in a sample of young adults and a sample of older adults.

The studies discussed above used intelligibility as the main metric. We have added complementary findings to this work through the use of multi-dimensional subjective feedback on conversation-level excerpts, rather than the short-duration sentences typically used. The task we used was short and the pattern of the ratings obtained suggest task demands were low. The task featured favourable listening conditions: speech was presented in quiet, all possible talkers were visible throughout, and even though the speech was natural, talkers were respectful of turn-taking, leading to little overlapping of speech. Yet, significant effects of the auditory variable were still observed. While relatively less problematic than the effect of removing visual cues, the use of face coverings was found to negatively impact each of the subjective aspects of communication measured. Even small effects like these can accumulate over extended periods, especially in less favourable listening conditions, requiring more cognitive resources and potentially leading to fatigue (Carraturo et al., 2023; McGarrigle et al., 2014).

Overall, in the trade-off between degree of acoustic attenuation and the restoration of the visual cues, the magnitude of the effects observed indicate that the introduction of a transparent window into face coverings should aim to result in less overall attenuation than that produced by FFP3-style masks, at least for speech in quiet for normal hearing participants. Better would be to bring the benefits afforded by increased visibility, without also incurring the costs associated with heavily attenuated transmission of speech. First steps along this path have resulted from the work of Cox et al. (2022), who report an open-source design for a “community face covering” (MakerSpace, 2022) that introduces a transparent panel that results in a low degree of attenuation, similar to that of a surgical mask.

A strength of the methodology used in the current work was that the same underlying recordings could be used for all stimuli, allowing for the greatest possible degree of control when attempting to isolate the effects of the experimental manipulations. Consequently, however, we were not able to account for any (deliberate or spontaneous) behavioural adjustments speakers may have made had they actually been wearing surgical masks, such as speaking more slowly or clearly, for example (McKenna et al., 2022). Our normalisation of audio level across all conditions would be expected to produce only small changes in overall level since the bulk of the filtering affected the high frequencies, which contribute little to the overall signal power. Although this level adjustment would partially mirror the real-world behavioural adjustment of talking more loudly when wearing a face covering, the relationship was not necessarily accurately portrayed in this experiment. Thus, while our results accurately reflect perceptual differences between controlled examples, they may not necessarily fully reflect real-world behaviour and perception of talkers when wearing different coverings. Similarly, for the visual variable, this study provides an estimate of the degree of impairment resulting loss of visual cues when wearing a surgical-style mask. It should be noted, however, that this is not the same as directly estimating the benefit associated with the introduction of a transparent panel to face coverings, as typically some portion of the lower face remains obscured, and other issues such as glare or fogging can arise. Another difference to note when interpreting these findings is the likely difference between the deliberately favourable listening conditions of the task and those regularly encountered in real-world interactions. Relatively low ratings for effort, alongside higher ratings for speech clarity and the amount of conversation followed, suggest that participants did not typically find the task particularly challenging. This finding also likely reflects the characteristics of the participant sample, which was relatively young and mostly reported normal hearing. An older population with hearing loss might have found the task more demanding. Additionally, although the conversation was entirely natural, those involved in it did not generally talk over one another, meaning instances of multiple talkers were rare. Recordings were made in the absence of background noise, but since teleconferencing uses perceptual coders for both audio and video data streams, already the streams are information-reduced. The amount of information removed will depend on factors beyond the control of the experiment such as the bitrate used by the coders. This can be expected to have some impact, especially on those with hearing impairment. Future expansion of the current work could focus on the relative contributions of audio-visual streams under more challenging listening conditions, and the inclusion of more hearing-impaired participants. Task difficulty could be increased by any combination of the introduction of background noise, stronger tests of comprehension of the content of stimuli, or the use of longer clips, for example. These adjustments would also bring in other real-world factors such as alertness and motivation. Our predicted interaction between audio and visual variables was not observed for two out of the three measures. One possibility is that the predicted differences would have been more apparent under less favourable listening conditions, as previous work suggests (Toscano & Toscano, 2021).

The emphasis on real-world communication in this study meant we had to record and edit custom-made stimuli and could not rely on existing test materials and their associated measurement scales. We used novel stimuli and measures, which allowed us to present natural, unscripted conversation and to gather information about participants’ subjective experiences of them. This approach also means there is a lack of evidence to support validity and reliability for the measures used; however, the data show face validity in that ratings were negatively impacted by the simulated attenuating effect of face coverings and by the presence of a mask covering the lips and lower part of the face. To maximise the transparency of our use of the novel measures, we pre-registered the number and wording of all questions presented to participants, as well as the scale endpoints and the underlying numerical values used. All measures were reported in full and without deviation from the preregistration.

Finally, discussion of trade-offs between design of face coverings and usage scenario should also acknowledge that the goal should be to inform decision-making about design and use of coverings, rather than an attempt to converge upon some single, optimal design. Trade-offs and preferences will vary per individual. In addition to the audio and visual cues manipulated in the current work, real-world outcomes for communication are a dynamic, complex interplay between a range of factors including the content and context of an interaction, listening environment and the level and type of background noise, the familiarity and type of a speech of a talker, and the hearing health of the listener (Yi et al., 2021, 2023). For example, as the level of background noise increases, or there are multiple simultaneous talkers, there is increased reliance on visual cues, and even small improvements in the relative transmission of the speech signal become increasingly important.

## Conclusion

Although the current task was performed in favourable listening conditions, such as without background noise and with little conversational overlap between talkers, and was completed by mostly younger normally-hearing listeners, significant effects on aspects of natural conversation, rather than just intelligibility, were induced by face coverings in both the acoustic and visual domains. The importance of these findings to the ongoing use of face coverings is not limited to pandemic situations. In healthcare settings, mask use remains widespread. Here, information transmitted and style of communication are more complex than in domestic settings, and so more sophisticated face covering designs are required so as to enable ease of communication (Saunders et al., 2020). Given that coverings always introduce attenuation, identifying the degree of attenuation that can be offset by restoration of visual cues is an important aspect of the overall design of the covering. Here we identified that degree for communication in a relatively easy listening environment. This degree can be expected to vary with listening environment such as with the introduction of background noise or distortion. Face covering design cannot be a “one-size-fits-all” approach. Our findings contribute to a stronger understanding of the effects of face covering design, helping to inform future design improvements and strategies for communication in environments where face coverings are required.

## Data Availability

Supplementary analyses, code for analysis, and de-identified data are openly available in the online repository for the project, which can be found at https://osf.io/r9tmh/. MATLAB code used for filtering audio is also available in this repository.

## Abbreviations

dB: Decibel
FFP3: Filtering face piece, class 3
N99: Non-oil, 99% (i.e. a mask which is 99% efficient at blocking airborne contaminants, in environments not containing oil-based particulates, similar in function to FFP3)
RMS: Root mean square
SD: Standard deviation
SNR: Signal-to-noise ratio
